# Rapid lipid bilayer membrane formation on Parylene coated apertures to perform ion channel analyses

**DOI:** 10.1007/s10544-020-0473-y

**Published:** 2020-04-30

**Authors:** Tanzir Ahmed, Sander van den Driesche, Jayesh Arun Bafna, Martin Oellers, Roland Hemmler, Karsten Gall, Richard Wagner, Mathias Winterhalter, Michael J. Vellekoop

**Affiliations:** 1 Institute for Microsensors, -actuators and -systems (IMSAS), University of Bremen, Microsystems Center Bremen (MCB), Bremen, Germany; 2 Department of Life Sciences and Chemistry, Jacobs University Bremen, Bremen, Germany; 3 Ionovation GmbH, Bissendorf, Germany

**Keywords:** Parylene-C, Parylene-AF4, Lipid Bilayer Membrane (LBL), Silicon, Silicon Nitride

## Abstract

We present a chip design allowing rapid and robust lipid bilayer (LBL) membrane formation using a Parylene coated thin silicon nitride aperture. After bilayer formation, single membrane channels can be reconstituted and characterized by electrophysiology. The ability for robust reconstitution will allow parallelization and enhanced screening of small molecule drugs acting on or permeating across the membrane channel. The aperture was realized on a microfabricated silicon nitride membrane by using standard clean-room fabrication processes. To ensure the lipid bilayer formation, the nitride membrane was coated with a hydrophobic and biocompatible Parylene layer. We tested both Parylene-C and Parylene-AF4. The contact angle measurements on both Parylene types showed very good hydrophobic properties and affinity to lipids. No precoating of the Parylene with an organic solvent is needed to make the aperture lipophilic, in contradiction to Teflon membranes. The chips can be easily placed in an array utilizing a 3D printed platform. Experiments show repetitive LBL formation and destruction (more than 6 times) within a very short time (few seconds). Through measurements we have established that the LBL layers are very thin. This allows the investigation of the fusion process of membrane proteins i.e. outer membrane protein (OmpF) in the LBL within a few minutes.

**Electronic supplementary material:**

The online version of this article (10.1007/s10544-020-0473-y) contains supplementary material, which is available to authorized users.

## Introduction

Membrane channels play a crucial role in controlling the permeability of cell membranes. Nutrients have to be taken up while cell toxic substances are blocked. This makes them a major target for pharmaceutical drug screenings and helps to identify the causes of various diseases (Dunlop et al. 2008). The functional behavior of ion channels can be investigated by recording their transmembrane ionic current (Hille 2001; Gutsmann et al. 2014; Williams 1994). Patch clamping is a widely used technique to measure the current being transported through the membrane-bounded pores upon an applied voltage (Sakmann et al. 1995). In patch clamping, a small region of the membrane of a living cell is patched in a narrow tip of a glass pipette filled with electrolyte solution whereas an additional integrated Ag/AgCl electrode (immersed in the electrolyte) is connected to an amplification unit for signal acquisition. However, patch clamping is time and cost intensive and easily leads to an unstable current limiting the possibility to detect single-channel events (Kongsuphol et al. 2013; Zagnoni 2012; Saha et al. 2014). Patch clamping can efficiently be implemented in relatively large multicellullar tissues or mammalian cells. In contrast, many more complexities will occur when dealing with smaller species like bacteria because of the high input resistance (more than 5 GOhm) (Wilson et al. 2011).

Biological cells are protected by a membrane consisting of phospholipids with a hydrophilic head and hydrophobic tails. Due to these properties, they spontaneously form a double layer where the hydrophobic tail groups are shielded by the hydrophilic head groups when suspended in an aqueous solution (Gennis 2013; Kramar et al. 2010). By utilizing this self assembly mechanism, a planar bilayer membrane can be formed in a lipophilic microaperture. It is a simple yet effective method to mimic a cellular membrane without the need of a living cell. An artificial bilayer platform requires an aperture punched in a foil, fixed between two fluid reservoirs, which are filled with an electrolyte solution (Kramar et al. 2010). Pore proteins (e.g. Outer membrane porin F and alpha-hemolysin) can be added and fused into the LBL in a controlled way. Its characteristic ion permeation through the pores can be investigated upon applying a DC potential over the two fluid reservoirs.

Formation of a LBL on a hydrophobic material in an aperture can be realized by utilizing various methods. A widely used technique to form such artificial bilayers is the painting method. It involves painting the lipid dissolved in the organic non-polar solvent (e.g. n-decane) by a paint brush or borosilicate glass rod on a hydrophobic sheet containing the aperture separating two fluid chambers. Lipid molecules will naturally arrange at the interface between the organic and aqueous phases on both sides of the aperture. The film will gradually thin down from the center thus constructing the bilayer spontaneously. During the thinning process, the excess solvent will slowly accumulate at the apertures edge creating a mechanically stable connection (solvent annulus: Plateau-Gibbs border to the bilayer (Zagnoni 2012; Fujiwara et al. 2003).

Alternatively, a LBL could be quickly realized by applying a pseudo painting method where an air bubble, coated by lipid molecules are used Braun et al. (2014). In this method, a pipette tip was precoated with the lipids dissolved in n-octane (e.g. n-octane). Afterward, this wetted pipette tip was slowly moved towards the aperture immersed in the buffer phase to create the air bubble covered with lipid molecules. Once the bubble bursts on the aperture wall, the lipid spontaneously rearranges forming a bilayer. Another approach is termed: solvent-free lipid bilayer formation, first introduced by Montal and Mueller (1972). Here, two lipid monolayers were created after spontaneous rearrangement of the lipid molecules at the air-water interface. Initially, the height of the air-water interface in both reservoirs are kept below the aperture location. Later, both buffer levels will be raised above the aperture, allowing the creation of a solvent-free lipid bilayer. Utilizing these above mentioned methods, an artificial cellular environment can be easily realized ensuring a tight sealing with a very low leakage current that is suitable for the detection of single channel events (Kramar et al. 2010).

## Problem description

The major advantage of a bilayer fixed in a vertically placed suspended aperture is that the membrane is easily accessible from both sides. This makes it possible to achieve an easy exchange of liquid solution, e.g. allowing asymmetric and inhomogeneous salt gradients or pH gradients across the LBL (Dunlop et al. 2008; Zagnoni 2012; Saha et al. 2014; Kramar et al. 2010). Current bilayer platforms use Teflon foils with an aperture realized by mechanical punching or laser ablation (Hanke and Schulue 2012; Hansen et al. 2009). However, Teflon needs to be precoated by non volatile organic solvent i.e. 1% (v/v) lipid dissolved in chloroform solution to make the material lipophilic. This requires additional processing time of 15 to 20 minutes to let the solvent evaporate (Gelder et al. 2000), which is a hindrance for pushing this technology for automated applications. Additionally, placing the Teflon foils in the array can be a time-consuming task.

Modern microfabrication techniques serve as an efficient alternative to realize precise, well defined planar hydrophobic apertures in various substrates. Recently Tadaki et al. reported a series of microfabrication steps to achieve a tapered microaperture on a thin silicon nitride membrane. The tapered geometry was realized by an isotropic etching step of silicon nitride. Unfortunately, it caused crack formation in the membrane (Tadaki et al. 2017). Handling such fragile chips could be quite problematic and assembling them in a parallel microfluidic platform would be a rather challenging task. To make a chip surface, which is coated with a fluoropolymer (CYTOP or Teflon) lipophilic, an additional pretreatment step with organic solvents is needed (Tadaki et al. 2017; Oshima et al. 2012).

Another tapered aperture fabrication step was demonstrated by Baker et al. to improve the mechanical stability of the formed bilayer. Despite the fact that it yields stable bilayers, it involves rather complex fabrication steps of implementing rotational UV exposure on the SU8 negative photoresist to define the aperture geometry (Baker et al. 2013).

Suzuki et al. reported on the horizontal integration of a 20 µm thick Parylene-C sheet with an array of micro-apertures in a 3D printed parallel platform realized by microstereolithography (Maruo et al. 2002; Suzuki et al. 2008). After the microfabrication, the Parylene sheet had to be peeled off from the wafer. The resultant is a flexible sheet that requires significant alignment efforts to the 3D platform. Also, the enclosed microchannel underneath, might be prone to air bubble sticking.

A recent work published by Rossi et al. (2012) demonstrated a parallel bilayer platform with an automated fluid dispensing unit. Using this automated approach, bilayers were created in a punched Teflon sheet followed by solvent-free bilayer formation (Montal and Mueller 1972). However, the lack of an annulus containing solvent results in a mechanically unstable bilayer (Zagnoni 2012).

## Proposed solution

To resolve the aforementioned issues and limitations, we present a highly reliable microfabricated silicon chip for rapid LBL formation. To ensure the biocompatibility and hydrophobicity, the chip surface was covered with a conformal polymer coating namely, Parylene-C or Parylene-AF4 respectively. Both materials showed very good lipophilic properties and do not require any precoating of organic solvent to obtain perfectly thinned membranes. For parallel bilayer formation, a 3D printed measurement platform realized by microstereolithography is introduced. A similar concept has already been presented in our previous work where the LBL was realized within a short time (30 to 130 seconds) (Ahmed et al. 2018). The 3D printed platform contains microfluidic reservoirs dedicated for individual chips, each block can accommodate four of them in a tilted position. Such tilted placement of the chips allows easier lipid injection possibilities for an automated fluid dispensing robot.

With the chips (coated with Parylene-AF4 or with Parylene-C) and the measurement platform, repetitive LBL construction (using pseudo air bubble painting) and destruction by applying a 1 Volt pulse was obtained within a few seconds. This confirms the realization of thin solvent-free membranes in a fast and reproducible way. Moreover, both chip types showed a similar LBL lifetime between 40 minutes to one hour, which is more than sufficient for electrophysiology measurements. Finally, successful incorporation of membrane protein OmpF (outer membrane protein F) in the LBL with its characteristic ion channel activities was demonstrated.

## Materials and methods

### Design and device fabrication

**Fig. 1:**
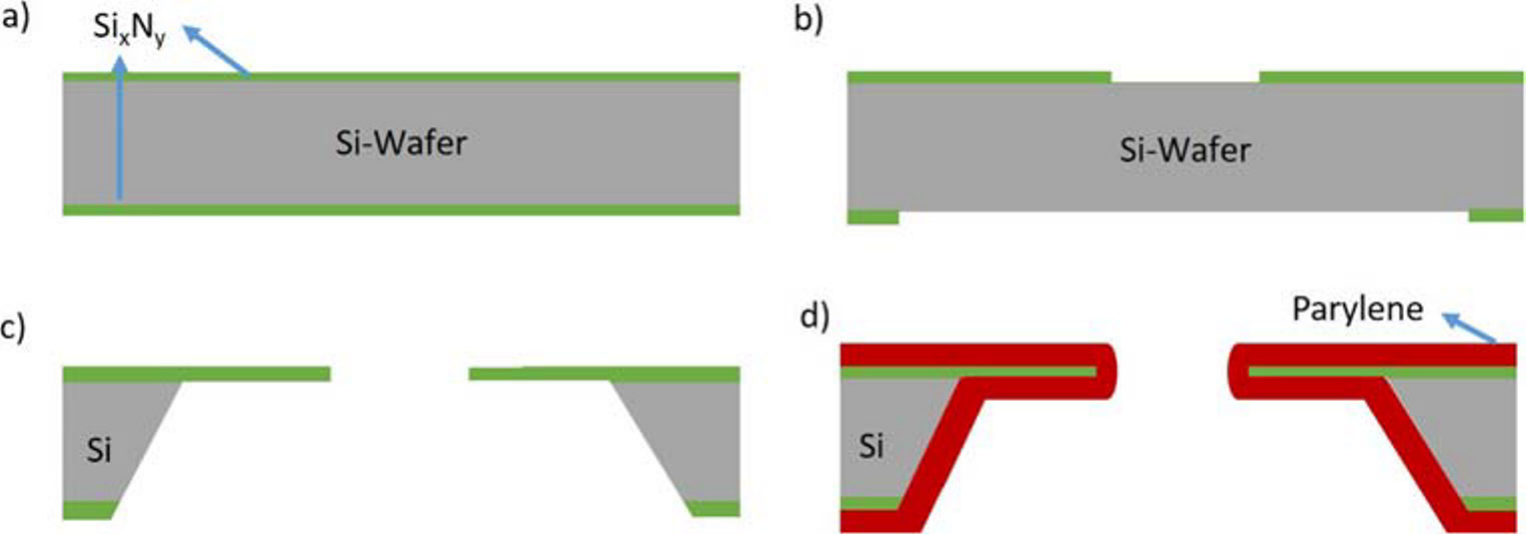
Fabrication steps of the chip **a** 500 nm Silicon Nitride deposition (Si_x_N_y_) **b** photolithography and reactive ion etching at front and back side of the wafer to transfer aperture and etching profile **c** anisotropic etching of silicon with 30% KOH at 80°C **d** 10 µm Parylene-C or 4 µm Parylene-AF4 deposition

The microfabrication of the silicon chips were conducted in a cleanroom environment. The fabrication steps are shown in Fig. 1a–d. A thin layer of silicon nitride (500 nm) was deposited at both sides of a double side polished silicon wafer of 380 µm thickness by using LPCVD (Fig. 1a). Afterwards, a photolithography step using positive photoresist AZ1518 was applied at both sides of the wafer for transferring the aperture pattern and the etching window, respectively (Fig. 1b). Reactive ion etching and subsequent development with resist remover (AZ 100) were conducted for 15 minutes to open the silicon underneath of nitride layer (Fig. 1c). To open the aperture from either side of the wafer, it was exposed to a 30% KOH solution at 80°C to induce anisotropic etching of silicon (Fig. 1d). Lastly, Parylene-C and Parylene-AF4 were chemically vapor deposited on two separate wafers to ensure the hydrophobic surface.

**Fig. 2:**
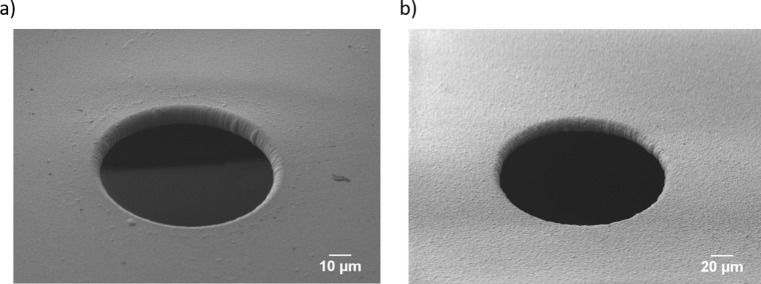
Parylene coated apertures **a** Parylene-C coated 100 µm aperture **b** Parylene-AF4 coated 200 µm aperture

Prior to the Parylene deposition, the wafer was exposed to adhesion promoter silane A-174 (methacryloxypropyltrimethoxysilane) to improve the attachment between the nitride and Parylene. The Silane A-174 (PPS, Germany) was suspended in deionized water and isopropyl alcohol (IPA, technical grade) with a volume ratio of 2 : 5 : 95 (Silane : DI-H_2_O : IPA). Afterwards, stirring of 30 minutes is required to hydrolyze the silane. Before placing the wafer in the A-174 solution, it was treated with peroxymonosulfuric acid for 15 minutes and later rinsed with IPA to create the hydroxyl (-OH) group on its surface. Next, the wafer was placed in A-174 solution for 5 minutes and then another rinsing with IPA was done to remove the non-adhered adhesion promoter. After drying and baking the wafer at 120°C for 2 minutes the surface should contain sufficient covalent bonds between the substrate and silane. Then the wafer was left for some hours to remove the water during covalent bond formation. The Parylene-C deposition was conducted in three stages. Initially, the solid dimers are vaporized at 150°C and later pyrolyzed to obtain diradical monomer at a high temperature process at 650°C. Finally, the monomeric vapor condensates at 50°C and deposited as a transparent, conformal coating on the substrate surface (Dolbier and Beach 2003; PPS Plasma Parylene Systems 2019). To achieve 10 µm and 4 µm coatings of Parylene-C and Parylene-AF4, 20 gram Parylene-C and 16 gram AF4 dimers were required. The two types of Parylene have different physical properties. Parylene-AF4 is less permeable for water compared to Parylene-C (Specialty Coating Systems 2020). Therefore, a thinner AF4 coating was used. In Fig. 2a, b, the SEM images of the fabricated microapertures after Parylene-C and AF4 deposition are depicted.

### The 3D printed chip holder and assembly

**Fig. 3:**
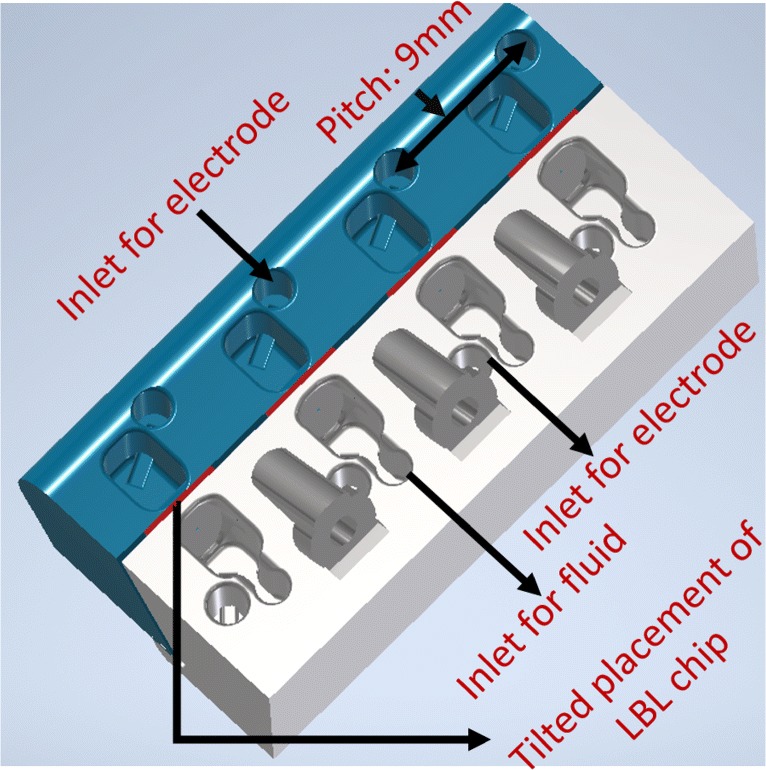
Design of the proposed 3D chip-holder depicting the assembling of the four LBL chips with tilted placement in the chip holder

The parallel bilayer measurement platform is designed in Autodesk Inventor 2018 software. As demonstrated in Fig. 3, the 3D design consists of two separate blocks with four parallel 40 µL fluid chambers. Each fluid reservoir has its own fluid and electrode access for the pipetting robot with a pitch distance of 9 mm. Instead of placing the chips vertically, the design offers a tilted chip placement to facilitate an easier lipid injection and protein loading by the pipetting robot depicted by the 3D model (Fig. 3). We have used medical grade double sided tape to fix the chips simply between two 3D printed blocks.

**Fig. 4:**
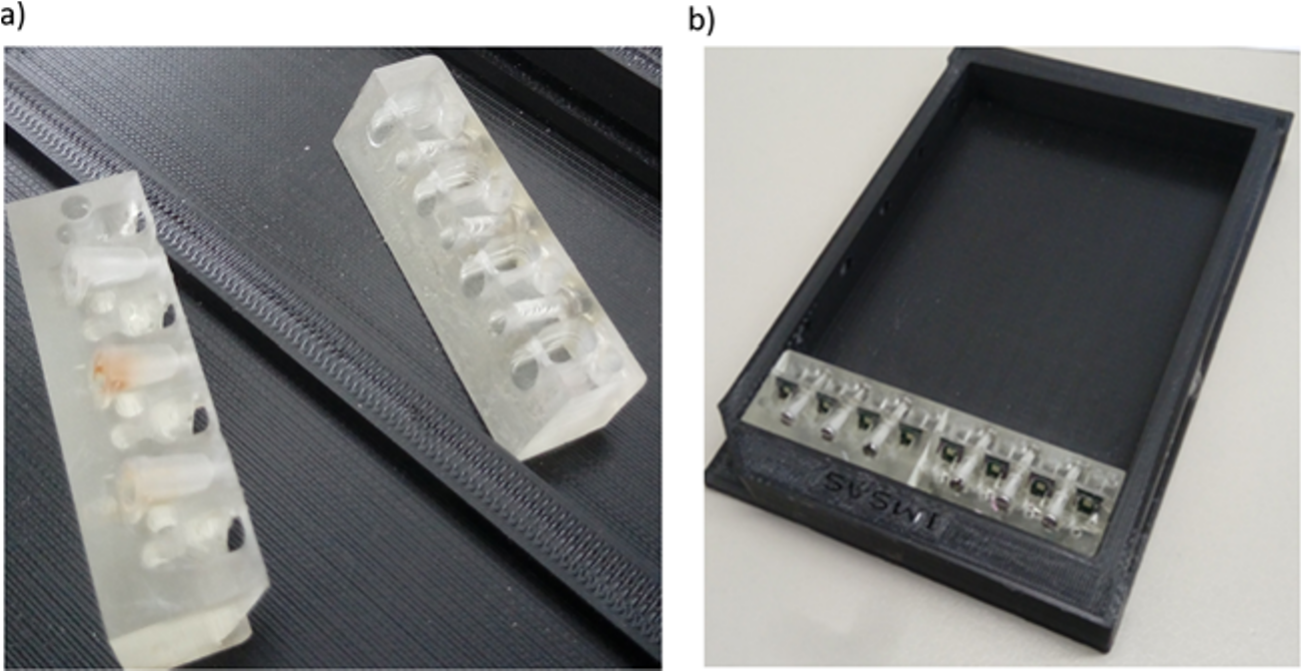
The 3D printed prototypes **a** a pair of 3D printed chip holders **b** assembly of the 3D printed chip holder containing lipid bilayer chips in a 96 well plate configuration

An Asiga MAX X27 (385 nm) 3D printer was used to print the chip holder. The printing material was a semitransparent photo curable resin (PlastCLEAR). The printer has a lateral resolution of 27 µm with a minimum slicing thickness of 10 µm. The build envelop is 51 x 29 x 76 mm^3^. After 3D printing of the model, it was removed from the printing platform and cleaned with Isopropanol. The model was placed in an UV chamber to crosslink the remaining uncured resin and dried with nitrogen gas (van den Driesche et al. 2018). A pair of 3D printed chip holders are shown in Fig. 4a. We printed four pairs of such aforementioned chip holders for conducting the experimental works. This chip holder design is compatible to be assembled in a 96 well configuration allowing parallel bilayer analyses by applying an automated pipetting robot (Fig. 4b).

### Bilayer formation

**Fig. 5:**
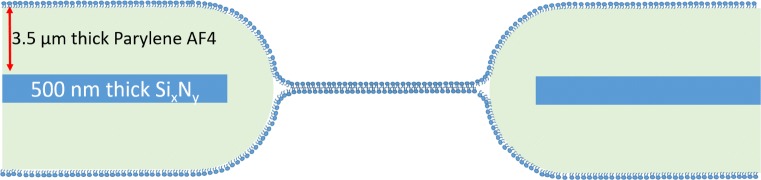
Schematic of a LBL formed in a Parylene-AF4 coated aperture (image is not drawn to scale)

After assembling the four chips in the chip holder, each reservoir was cleaned with ethanol, deionized water and dried with nitrogen gas. The bilayer was construction by painting air bubbles coated with lipid solution (1,2-diphytanoyl-sn-glycero-3-phosphocholine, DPhPC, diluted in octane at a concentration of 5 mg/mL) on the aperture. The bilayer capacitance was measured by a current amplifier (eONE, Elements s.r.l., Italy) connected to Ag/AgCl electrodes placed at each fluid reservoir (cis and trans side of the LBL). The reservoirs were filled with 1 Molar NaCl solution and a 200 mV_pp_ triangular voltage was applied. Without the presence of a bilayer, the resultant peak saturation current is 20 nA, meaning that both chambers are electrically connected and that the aperture is ready to be painted with lipid. Upon successful bilayer formation, the electric signal was recorded at a sampling frequency of 10 kHz. A schematic of a lipid membrane in a Parylene coated aperture is depicted (Fig. 5).

## Results and discussion

### Contact angle measurements of Parylene coatings

**Fig. 6:**
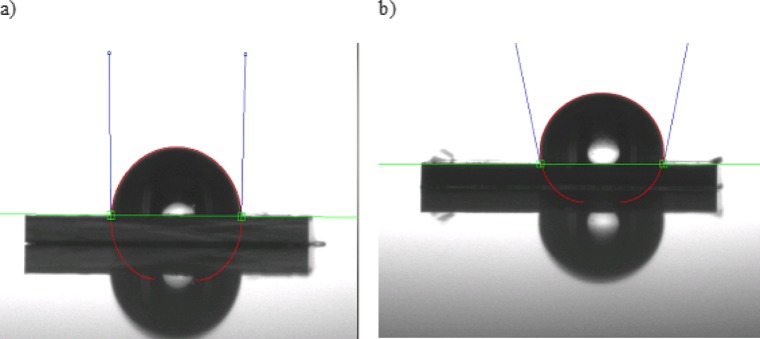
Static water droplet contact angle measurement **a** on a Parylene-C surface **b** on a Parylene-AF4 surface

The hydrophobicity of Parylene-C and Parylene-AF4 coated surfaces was determined by measuring the contact angle of a water droplet and a lipid droplet placed on the substrate. In case of Parylene-C, the measured static contact angle is 92° whereas the contact angle of the Parylene-AF4 coated surface is 101°, see Fig. 6a, b.

**Fig. 7:**
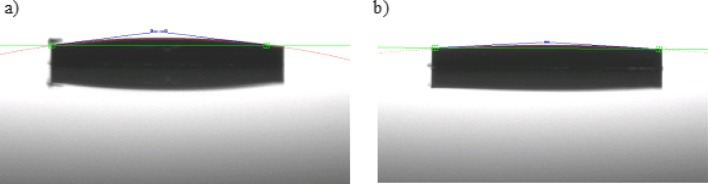
Contact angle measurement of a 1µL lipid solution droplet **a** on a Parylene-C surface **b** on a Parylene-AF4 surface

The following images (Fig. 7a, b) show that both types of Parylene coated surfaces have very good lipophilic properties. The measured contact angle for a droplet of 1 µL lipid solution is 7° and 3° respectively for Parylene-C and AF4 coated surface.

### Thickness measurements of Parylene coatings

**Fig. 8:**
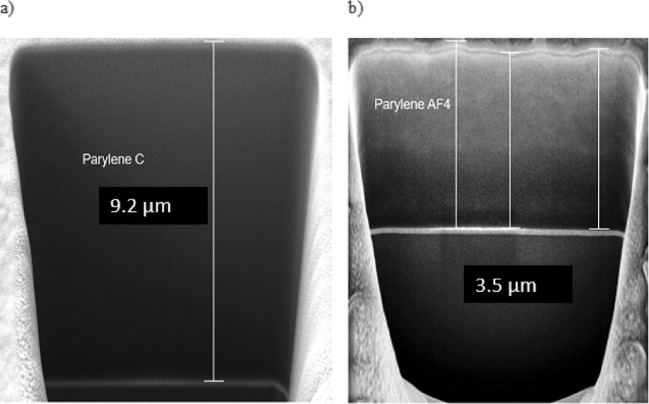
Thickness measurement of Parylene. The recess was cut with a high energized focused ion beam **a** measured Parylene-C thickness is 9.2 µm and Parylene-AF4 using SEM imaging. **b** measured Parylene-AF4 thickness is 3.5 µm and Parylene-AF4 using SEM imaging

In order to measure the Parylene thickness after deposition, a small window was cut in the layer with a high energized focused ion beam (FIB), on a chip surface. The thickness was measured by scanning electron microscopy (SEM). For this purpose, a cross beam (FIB-SEM) workstation from Carl Zeiss (Auriga Series 40) was used. To avoid the charging effect during SEM imaging, a thin layer of gold was sputtered. To cut a 10 x 20 µm^2^ window in the chip, a beam energy of 30 kV with 1 nA probe current was applied. As depicted in Fig. 8a, b, the measured thickness of the Parylene-C and AF4 amounts to 9.2 µm and 3.5 µm respectively.

### Electrical LBL measurements

**Fig. 9:**
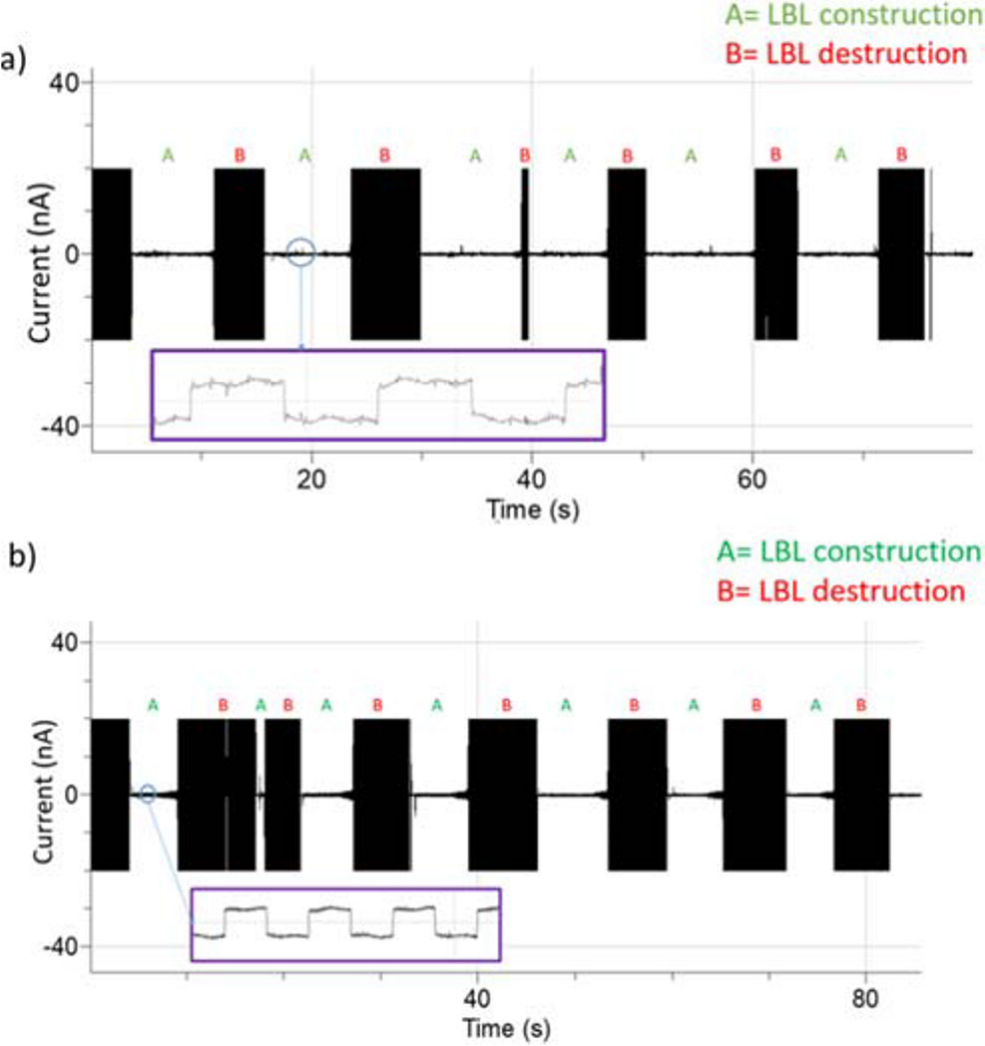
Repetitive LBL construction using the pseudo painting method **a** on a 80 µm Parylene-AF4 chip aperture and subsequent destruction (n = 6 consecutive times) by applying 1 volt pulse **b** LBL construction and destruction cycle (n = 7 consecutive times) after forming the membrane in a 90 µm Parylene-C coated aperture. The inserts show the corresponding current signals after LBL formation

For fast and reproducible protein fusion experiments, it is important that the constructed LBL is properly thinned. A common method to check this is by applying a DC voltage (ca. 1 Volt) over the LBL. A thinned membrane should break instantly upon applying such DC voltage. In contrast, a thicker membrane sould not break due to solvent clogging or accumulation of multiple lipid layers (Braun et al. 2014). In this work, repetitive LBL construction and destruction have been realized in both chip types, confirming the quality of successfully thinned membranes suspended in the Parylene coated apertures. In Fig. 9a, b, repetitive cycles of LBL construction and destruction for a Parylene-AF4 and Parylene-C coated chips (n = 6 and n = 7 times) are shown. An individual cycle was typically achieved within a few seconds.

The bilayer can be modeled as a parallel plate capacitor and the resultant capacitance can be mathematically derived for a specific aperture diameter (Eq. 1). Here, the capacitance (C_bilayer_) is a function of the area *A* of the bilayer while assuming the dielectric constant of the hydrophobic chain *𝜖*_*lipid*_ is 2.1 and the thickness *d* (Hille 2001). The permittivity of the free space is given by, *ε*_0_ = 8.854x10^-12^ Fm^-1^. Therefore, the bilayer capacitance equation can be written as,

1 $$ C_{bilayer}=\epsilon_{lipid}\epsilon_{0}A/d  $$

The resultant capacitance can be expressed as per unit area (C_s_) of the aperture. For example, if a phospholipid tail thickness of 2.4 nm is assumed (Hille 2001), a 90 µm aperture would yield a LBL capacitance of 47 pF. Practically, the C_s_ of the LBL formed by DPhPC lipid dissolved in organic solvent, is in the range of 4.2 to 13 fF/µm^2^. Results obtained from following parallel LBL formation experiments demonstrated consistent C_s_ compared to the values found in the literature (Kramar et al. 2010; Costa et al. 2013; Scalas et al. 1998; Ridi et al. 1998).

To realize the LBLs in parallel, four 3D printed blocks were prepared containing different chip designs for conducting sixteen measurements. In the first two blocks, eight Parylene-C chips were mounted (each block: two chips with 90 µm aperture, and two with 70 µm aperture) and the remaining two blocks were prepared with eight Parylene-AF4 coated chips (each block: four chips with 80 µm aperture) to check the possibilities of LBL formation. In this work, only single 3D printed blocks were used at each trails during the experiment because of the limited instrumentation.

#### Parylene-C chips: Block-1 and Block-2

After LBL construction on the 90 µm and 70 µm chip aperture in block-1 the capacitance was measured: 43 to 50 pF and 30 to 33 pF, respectively. By using these capacitance values in equation 1, the calculated thickness of the phospholopid tails amounts to 2.4 nm to 2.7 nm confirming the existence of the thin synthetic suspended membrane. In both blocks, the LBL stays intact between at least forty minutes to one hour, whereas in Teflon it lasts between three to five minutes (Braun et al. 2014). Therefore, the formed LBL lifetime is more than enough to investigate the ion channel activities. Considering both blocks, the resultant C_s_ of the LBL chip having 90 µm aperture is varied between 6 fF/µm^2^ to 7.8 fF/µm^2^ receptively. In case of the LBL chip with 70 µm aperture, the obtained C_s_ is in the range of 7.5 fF/µm^2^ to 9.8 fF/µm^2^.

#### Parylene-AF4 chips: Block-3 and Block-4

On the other hand, the LBL capacitance of Parylene-AF4 chips in block-3 ranged 35 to 48 pF. Here, equation 1 can be employed again to calculate the thickness *d* providing a value between 2.1 nm to 2.7 nm (C_s_ is between 7 fF/µm^2^ to 9.5 fF/µm^2^). However, a much lower LBL capacitance of 25 pF was observed in one of the chambers depicting a slightly thicker membrane (3.7 nm). In block-4, the resultant LBL capacitance was 29 pF to 38 pF (C_s_: 5.6 fF/µm^2^ to 7.6 fF/µm^2^) receptively. Here, a similar LBL lifetime was observed as compared to Parylene-C coated chips.

### Ion channel analysis

**Fig. 10:**
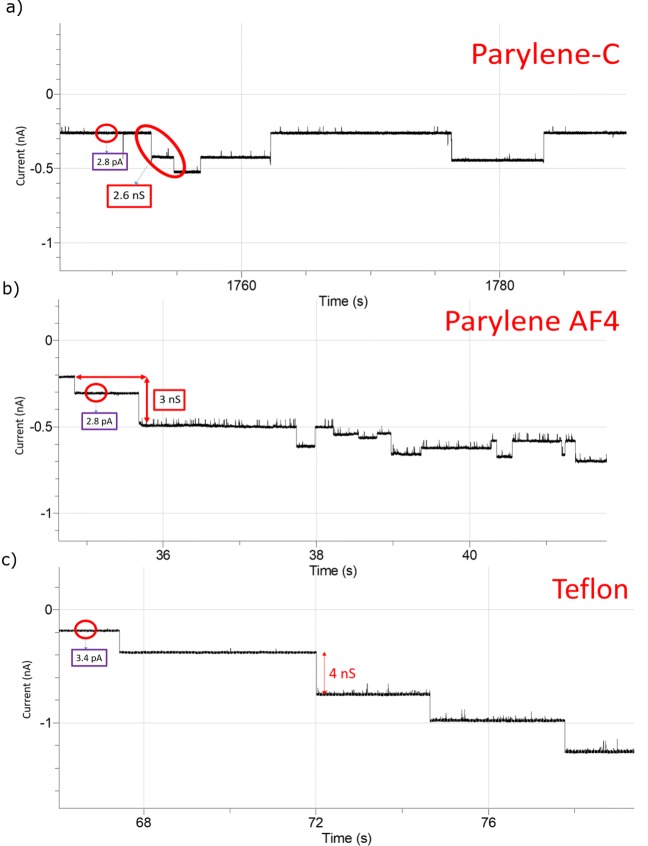
Current traces after protein (OmpF) insertion at -100 mV holding potential for the bilayer formed in **a** Parylene-C coated chip with 90 µm aperture and steady state unfiltered RMS noise of the current is 2.8 pA **b** Parylene-AF4 coated chip with 80 µm aperture (RMS value 2.8 pA). **c** Teflon aperture diameter approximately 100 µm (RMS value 3.4 pA)

To perform ion channel analysis, the experimental setup was placed in a shielded Faraday cage to reduce the noise level. The functionality of the created free standing LBL was tested by incorporating of OmpF with Parylene-C, AF4 and a Teflon aperture. In the experiment, DPhPC based lipid was used. It has been shown that even in supported arrangement, this lipid forms bilayers that remain in the viscous liquid crystalline (i.e. fluid) phase over a large temperature range (between -120°C and 120°C) (Kara et al. 2017). In previous works, temperature dependency of lipid diffusion within the bilayer has been investigated in large detail by FCS (fluorescence correlation spectroscopy), FCCS (fluorescence cross-correlation spectroscopy), and dual focus FCS measurements (Bartsch et al. 2012; Weiß and Enderlein 2012). Single-molecule tracking experiments of a ternary lipid mixture consisting of DPhPC:DOPC:DPPE (respectively: 1,2-diphytanoyl-sn-glycero-3-phosphocholine, 1-palmitoyl-2- oleoyl-sn-glycero-3-phosphocholine, 1,2-dipal-mitoyl-sn-glycero-3-phosphoethanolamine) at a molar ratio of 8:1:1, with either Atto647N-labelled DPhPC or labelled DPPE, were conducted at 25°C (Supplementary Material). The investigation clearly shows that lipid mobility in this free standing lipid bilayer was completely preserved. OmpF was extracted from *Escherichia coli* (stock solution 1 mg/ml OmpF) solution from the outer cellular wall of (*Escherichia coli*). In this work, this stock solution was diluted with detergent (genapol) by a factor of 10 as the genapol molecules attach to the hydrophobic area of the OmpF trimeric porins thus forming micelles (Gutsmann et al. 2014; Pebay-Peyroula et al. 1995). Prior to protein introduction, a 1 Volt pulse was applied to destroy the bilayer and reconstruction by pseudo painting to confirm the properly formed membrane. Here, 1 Molar KCl buffer (pH 7) was used in both chambers. After reforming the bilayer in a 90 µm Parylene-C coated aperture, 1.5 µL of diluted OmpF solution was added in the cis chamber. To insert the OmpF into the membrane, several smaller pump cycles by pipette were performed (Gutsmann et al. 2014). Within a few minutes ion channel activities took place at -100 mV DC potential as demonstrated in Fig. 10a. The intermediate current jumps of 260 pA shows the closure of the OmpF monomers (similar to Ahmed et al. 2018; Mahendran et al. 2010). A similar outcome was also observed at -100 mV with the Parylene-AF4 coated chip (80 µm aperture) where the resultant conductance was 3 nS as depicted in Fig. 10b. In case of a Teflon aperture (approximately 100 µm), similar trimeric closing events were also monitored while the DC potential was switched to -100 mV, see Fig. 10c. The highlighted current step yields a conductance level that amounts to 4 nS that is consistent with the value mentioned in the literature (Mahendran et al. 2010). Moreover, the RMS noise level of the steady unfiltered current signals are similar between the Parylene chips (2.8 pA) and the Teflon aperture (3.4 pA). However, the formation of a lipid membrane in Teflon required a time consuming pretreatment step to perform the measurement.

## Conclusions

This study shows that Parylene-C and -AF4 conformal-coated chips are highly suitable for fast (few seconds) and reproducible LBL formation. Compared to Teflon or other fluoropolymer based LBL supports, Parylene coated chips do not require time-consuming precoating steps with organic solvents to make the aperture lipophilic. Contact angle measurements demonstrate the lipophilic properties for both Parylene types. The high quality of the spanned LBLs is shown by repetitive LBL formation and destruction experiments. These confirm the realization of properly thinned bilayers using the LBL pseudo painting method. To verify our claim in terms of forming functional bilayers, pore containing protein OmpF was successfully incorporated to observe the ion channel activities. The trimeric conductance steps of OmpF shows its successful integration in the LBL.

## Electronic supplementary material

(DOCX 109 KB)

(MOV 18.2 MB)

## References

[CR1] Ahmed T, van den Driesche S, Oellers M, Hemmler R, Gall K, Bhamidimarri SP, Winterhalter M, Wagner R, Vellekoop MJ (2018). Proceedings.

[CR2] Baker CA, Bright LK, Aspinwall CA (2013). Anal. Chem..

[CR3] Bartsch P, Walter C, Selenschik P, Honigmann A, Wagner R (2012). Materials.

[CR4] Braun CJ, Baer T, Moroni A, Thiel G (2014). J. Neurosci. Methods.

[CR5] Costa JA, Nguyen DA, Leal-Pinto E, Gordon RE, Hanss B (2013). PLoS ONE.

[CR6] Dolbier WR, Beach WF (2003). J. Fluorine Chem..

[CR7] Dunlop J, Bowlby M, Peri R, Vasilyev D, Arias R (2008). Nat. Rev. Drug Discovery.

[CR8] Fujiwara H, Fujihara M, Ishiwata T (2003). J. Chem. Phys..

[CR9] Gelder PV, Dumas F, Winterhalter M (2000). Biophys. Chem..

[CR10] Gennis RB (2013). Biomembranes.

[CR11] Gutsmann T, Heimburg T, Keyser U, Mahendran KR, Winterhalter M (2014). Nat. Protoc..

[CR12] Hanke W, Schulue WR (2012). Planar Lipid Bilayers: Methods and Applications (Biological Techniques Series).

[CR13] Hansen JS, Perry M, Vogel J, Vissing T, Hansen CR, Geschke O, Emnéus J, Nielsen CH (2009). J. Micromechanics Microengineering.

[CR14] Hille B (2001). Ion Channels of Excitable Membranes.

[CR15] Kara S, Afonin S, Babii O, Tkachenko AN, Komarov IV, Ulrich AS (2017). Biochim. Biophys. Acta - Biomembr..

[CR16] Kongsuphol P, Fang KB, Ding Z (2013). Sens Actuators, B.

[CR17] P. Kramar, D. Miklavčič, M. Kotulska, A.M. Lebar, in . Advances in Planar Lipid Bilayers and Liposomes. 10.1016/s1554-4516(10)11002-3, Vol. 11 (Elsevier, 2010), pp. 29–69

[CR18] Mahendran KR, Kreir M, Weingart H, Fertig N, Winterhalter M (2010). J. Biomol. Screening.

[CR19] S. Maruo, K. Ikuta, T. Ninagawa, in . Technical Digest. MEMS 2001. 14th IEEE International Conference on Micro Electro Mechanical Systems (Cat. No.01CH37090). 10.1109/memsys.2001.906501 (IEEE, 2002), pp. 151–154

[CR20] Montal M, Mueller P (1972). Proc. Natl. Acad. Sci..

[CR21] Oshima A, Hirano-Iwata A, Nasu T, Kimura Y, Niwano M (2012). Micro and Nanosystems.

[CR22] Pebay-Peyroula E, Garavito R, Rosenbusch J, Zulauf M, Timmins P (1995). Structure.

[CR23] Ridi A, Scalas E, Robello M, Gliozzi A (1998). Thin Solid Films.

[CR24] Rossi M, Thei F, Tartagni M (2012). Sensors & Transducers.

[CR25] Saha SC, Thei F, de Planque MR, Morgan H (2014). Sens Actuators, B.

[CR26] B. Sakmann, E. Neher, 2 (eds.), *Single-Channel Recording* (Plenum Press, New York, 1995)

[CR27] Scalas E, Ridi A, Robello M, Gliozzi A (1998). EPL (Europhysics Letters).

[CR28] Suzuki H, Pioufle BL, Takeuhci S (2008). Biomed. Microdevices.

[CR29] PPS Plasma Parylene Systems, Accessed 18 Dec 2019. https://www.plasmaparylene.de/ (2019)

[CR30] Specialty Coating Systems, SCS parylene properties. https://scscoatings.com/corporate/technical-library/ (2020)

[CR31] D. Tadaki, D. Yamaura, S. Araki, M. Yoshida, K. Arata, T. Ohori, K. ichi Ishibashi, M. Kato, T. Ma, R. Miyata, Y. Tozawa, H. Yamamoto, M. Niwano, A. Hirano-Iwata, Sci. Rep. **7**(1). 10.1038/s41598-017-17905-x (2017) 10.1038/s41598-017-17905-x PMC5735097 29255199

[CR32] van den Driesche S, Lucklum F, Bunge F, Vellekoop M (2018). Micromachines.

[CR33] Weiß K, Enderlein J (2012). ChemPhysChem.

[CR34] Williams AJ (1994). Microelectrode Techniques, The Plymouth Workshop Handbook, chap. Chapter 5: An Introduction to the Methods Available for Ion Channel Reconstitution.

[CR35] Wilson JR, Clark RB, Banderali U, Giles WR (2011). Channels.

[CR36] Zagnoni M (2012). Lab. Chip.

