# Supplementary material for: Rapid lipid bilayer membrane formation on Parylene coated apertures to perform ion channel analyses
Source: Biomed Microdevices. 2020 Apr 30;22(2):32. doi: 10.1007/s10544-020-0473-y (PMC7192868; doi:10.1007/s10544-020-0473-y)
Supplement: Supplementary file 1 — (DOCX 109 KB) [file 10544_2020_473_MOESM1_ESM.docx]

**[Supplementary material](https://link.springer.com/article/10.1007/s10544-019-0450-5#SupplementaryMaterial)**

**Rapid lipid bilayer membrane formation on Parylene coated apertures to perform ion channel analyses**

Tanzir Ahmed^1^, Sander van den Driesche^1^, Jayesh Arun Bafna^3^ Martin Oellers^1^, Roland Hemmler^2^, Karsten Gall^2^, Richard Wagner^3^, Mathias Winterhalter^3^, and Michael J. Vellekoop^1^

^1^Institute for Microsensors, -actuators and -systems (IMSAS), University of Bremen, Microsystems Center Bremen (MCB), Bremen, Germany

^2^Ionovation GmbH, D-49143, Bissendorf, Germany

^3^Department of Life Sciences & Chemistry, Jacobs University, D-28759 Bremen, Germany.

**Single molecule tracking**

Single-molecule tracking experiments in free standing horizontal bilayers were performed on a fully motorised, infinity corrected Olympus IX 81 microscope equipped with a continuous wave (633 nm) HeNe laser (model nr. 31809, Research Electro Optics, Inc., Boulder, Colorado, USA) with a net power of 30 mW. Emitted light is collected through the same objective the excitation beam passes. After transmitting the dichroic beam splitter, it is reflected in a 90° angle to the incident beam by a mirror and finally projected onto the CCD-chip (of an electron multiplying charge-coupled device (EMCCD)), an air-cooled (-65 °C), back-illuminated camera (ImagEM, model nr. C9100-13, Hamamatsu Photonics, Hamamatsu City, Japan). The maximum quantum efficiency of the EMCCD camera is 90%. The magnification level of 60 resulted in an effective pixel size of 0.27 μm. Moreover, the CCD-array dimensions are 512 x 512 pixels. At full frame conditions, the shortest acquisition time is 30.5 ms. Data acquisition was performed using the software Hokawo and HCImage (both Hamamatsu Photonics), generating convertible image stacks.

Experiments were conducted at 25°C with a ternary lipid mixture consisting of DPhPC:DOPC:DPPE (respectively, 1,2-diphytanoyl-sn-glycero-3-phosphocholine, 1-palmitoyl-2-oleoyl-sn-glycero-3-phosphocholine, 1,2-dipal-mitoyl-sn-glycero-3-phosphoethanolamine) at a molar ratio of 8:1:1. With either Atto647N-labeled DPhPC or Atto647N-labelled DPPE. The determined capacitance of these lipid bilayer was 0.8 μF/cm² - 0.9 μF/cm² in good agreement with published data (typically 0.6 μF/cm²) [1]. The data of every single trajectory (see video) were fitted according to the one-component MLE model [2]. Every tracked molecule was evaluated for all time lags, which met the condition:

${time lag}_{max}=\left( \frac{individual framenumber}{10} \right)\cdot t_{lag}$ (1)

and a cumulative histogram of all determined diffusion coefficients $\bar{D}$ corresponding to all molecules investigated was created. As predicted from theoretical analysis [3], the histogram follows a gamma distribution with a mean of $\bar{D}= 11.9 \frac{\mu m^{2}}{s}$ and standard deviation 𝜎 = 2.1 $\frac{\mu m^{2}}{s} ($**Figure S1**).


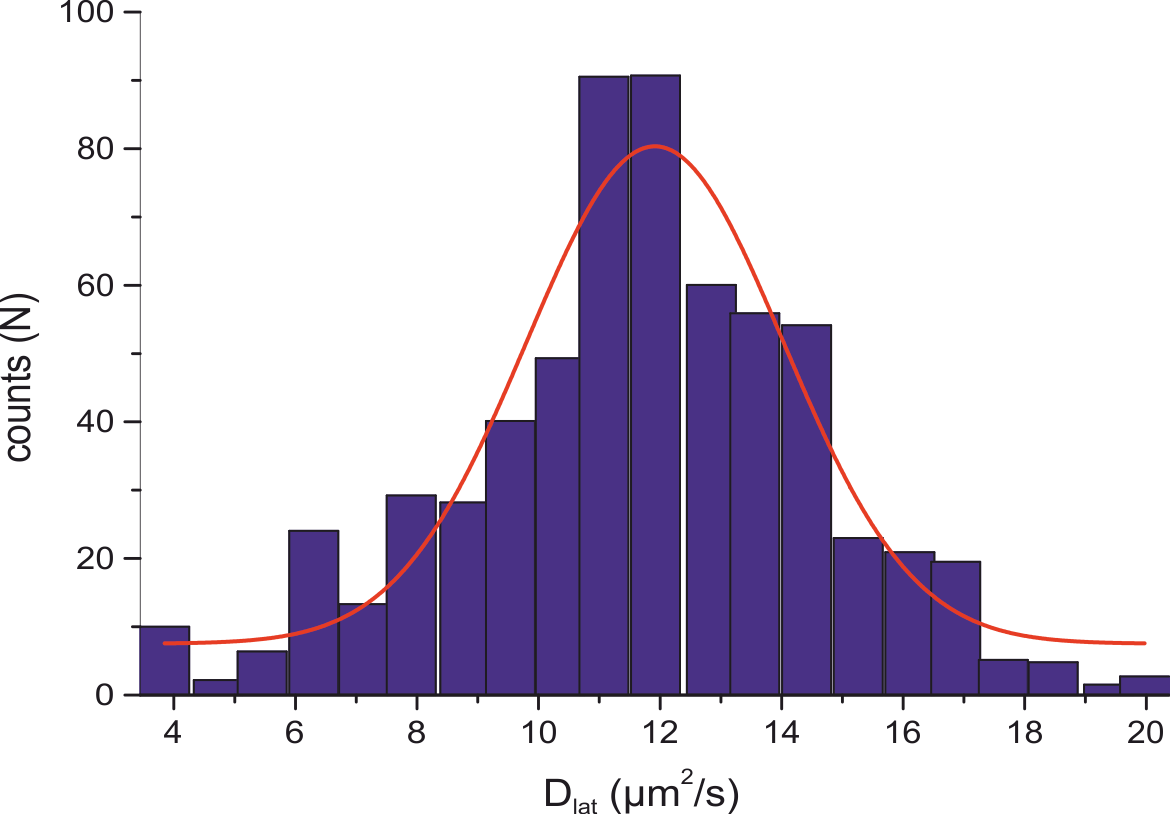


**Figure S1:** Left: Cumulative histogram of diffusion coefficients for all tracked molecules diffusing in a freestanding lipid bilayer. The distribution is characterized by a mean of 11.9 μm²/s and a standard deviation 𝜎 = 2.1 μm^2^/s.

Figure S1 shows the distribution of diffusion coefficients for measurements performed on free standing horizontal lipid bilayer containing in average roughly 10 labelled lipid molecules (Atto647N-DPhPC) within the circular recording area (r = 40µm). Similar results were obtained in the case of Atto647N-DPPE with $\bar{D}=13.9\frac{\mu m^{2}}{s} \mathrm{and}\sigma= 2.1 \mu\frac{\mu m^{2}}{s}$ .

These results show that at the given conditions both lipids can seemingly perform free diffusion similar to what has been observed with free-standing bilayer of comparable composition using the FCS technique and within living cells [4–7].

**References**

[1] H. Fujiwara, M. Fujihara, T. Ishiwata, Dynamics of the spontaneous formation of a planar phospholipid bilayer: A new approach by simultaneous electrical and optical measurements, J. Chem. Phys. 119 (2003) 6768–6775. doi:10.1063/1.1605372.

[2] A. Sonnleitner, G.J. Schütz, T. Schmidt, Free Brownian Motion of Individual Lipid Molecules in Biomembranes, Biophys. J. 77 (1999) 2638–2642. doi:10.1016/S0006-3495(99)77097-9.

[3] H. Qian, M.P. Sheetz, E.L. Elson, Single particle tracking. Analysis of diffusion and flow in two-dimensional systems, Biophys. J. 60 (1991) 910–921. doi:10.1016/S0006-3495(91)82125-7.

[4] K. Weiß, A. Neef, Q. Van, S. Kramer, I. Gregor, J. Enderlein, Quantifying the Diffusion of Membrane Proteins and Peptides in Black Lipid Membranes with 2-Focus Fluorescence Correlation Spectroscopy, Biophys. J. 105 (2013) 455–462. doi:10.1016/j.bpj.2013.06.004.

[5] A. Honigmann, C. Walter, F. Erdmann, C. Eggeling, R. Wagner, Characterization of Horizontal Lipid Bilayers as a Model System to Study Lipid Phase Separation, Biophys. J. 98 (2010) 2886–2894. doi:10.1016/j.bpj.2010.03.033.

[6] C. Eggeling, C. Ringemann, R. Medda, G. Schwarzmann, K. Sandhoff, S. Polyakova, V.N. Belov, B. Hein, C. von Middendorff, A. Schönle, S.W. Hell, Direct observation of the nanoscale dynamics of membrane lipids in a living cell, Nature. 457 (2009) 1159–1162. doi:10.1038/nature07596.

[7] P. Bartsch, C. Walter, P. Selenschik, A. Honigmann, R. Wagner, Horizontal Bilayer for Electrical and Optical Recordings, Materials (Basel). 5 (2012) 2705–2730. doi:10.3390/ma5122705.
